# Novel insights on acetylcholinesterase inhibition by *Convolvuluspluricaulis*, scopolamine and their combination in zebrafish

**DOI:** 10.1007/s13659-022-00332-5

**Published:** 2022-02-25

**Authors:** Kalyani Bindu Karunakaran, Anand Thiyagaraj, Kirankumar Santhakumar

**Affiliations:** 1 Supercomputer Education and Research Centre, Indian Institute of Science, Bengaluru, India; 2 Department of Genetic Engineering, SRM Institute of Science and Technology, Kattankulathur, 603 203 India; 3 Zebrafish Genetics Laboratory, Department of Genetic Engineering, SRM Institute of Science and Technology, Kattankulathur, 603 203 India

**Keywords:** Alzheimer’s disease, Acetylcholinesterase, Zebrafish, *Convolvulus pluricaulis*, Scopolamine, Isoxazole

## Abstract

Acetylcholinesterase (AChE) inhibitors increase the retention of acetylcholine (ACh) in synapses. Although they alleviate cognitive deficits in Alzheimer’s disease, their limited benefits warrant investigations of plant extracts with similar properties. We studied the anti-AChE activity of *Convolvulus pluricaulis* (CP) in a zebrafish model of cognitive impairment induced by scopolamine (SCOP). CP is a perennial herb with anti-amnesiac and anxiolytic properties. It contains alkaloid, anthocyanin, coumarin, flavonoid, phytosterol and triterpenoid components. Isoxazole (ISOX) was used as a positive control for AChE inhibition. CP-treated 168 hpf larvae showed a similar pattern of AChE inhibition (in the myelencephalon and somites) as that of ISOX-treated larvae. CP was superior to ISOX as evidenced by the retention of avoidance response behavior in adult zebrafish. Molecular docking studies indicated that ISOX binds Ser203 of the catalytic triad on the human AChE. The active components of CP—scopoletin and kaempferol—were bound by His447 of the catalytic triad, the anionic subsite of the catalytic center, and the peripheral anionic site. This suggested the ability of CP to mediate both competitive and non-competitive modes of inhibition. Surprisingly, SCOP showed AChE inhibition in larvae, possibly mediated via the choline-binding sites. CP + SCOP induced a concentration-dependent increase in AChE inhibition and ACh depletion. Abnormal motor responses were observed with ISOX, CP, ISOX + SCOP, and CP + SCOP, indicative of undesirable effects on the peripheral cholinergic system. Our study proposes the examination of CP, SCOP, and CP + SCOP as potential AChE inhibitors for their ability to modulate cognitive deficits.

**Supplementary Information:**

The online version contains supplementary material available at 10.1007/s13659-022-00332-5.

## Introduction

Alzheimer’s disease (AD) is a progressive neurodegenerative disorder characterized by memory loss, behavioral changes, and impaired cognition and language [1]. Around two-thirds of dementia cases have been attributed to AD, and it has an estimated prevalence of 10–30% in the population aged 65 years and more [1]. AD has a long pre-clinical phase of around 20 years and the average survival time for a person diagnosed with the disease is 8–10 years [2]. The two most distinctive hallmarks of AD are the accumulation of amyloid-beta plaques in the brain and the aggregation of tau proteins into neurofibrillary tangles within neurons [1]. Accumulation of amyloid-beta plaques leads to widespread non-specific degeneration of neurons, which in turn, affects various neurotransmitter systems including the cholinergic, monoaminergic, and glutamatergic systems [1]. Cognitive deficits seen in AD such as impaired memory and learning are often attributed to depleted levels of the neurotransmitter acetylcholine (ACh) in the basal forebrain cholinergic system, which is involved in arousal, memory coding, and storage and retrieval of working memory [3]. Therefore, maintaining substantial levels of ACh in the neuronal synapses of the cholinergic system may be integral to rescuing these cognitive deficits [4].

Depleted ACh levels could result from the dramatic loss of cholinergic neurons in the basal forebrain, reduced cholinergic innervation to the hippocampus and neocortex, reduced levels of the enzyme choline acetyltransferase (ChAT), and increased activity of the enzyme acetylcholinesterase (AChE) [2, 5–8]. AChE and ChAT modulate the levels of ACh in the central and peripheral cholinergic systems [2]. AChE catalyzes the breakdown of ACh into acetate and choline, while ChAT synthesizes ACh from choline and acetyl-CoA. ACh catabolism mediated by AChE serves two purposes. Firstly, it allows ACh to be continually replenished through the reuptake of choline into the synaptic knob. Secondly, it prevents neuronal hyperexcitability arising from enhanced ACh levels at the neuronal synapse [9]. However, excessive AChE activity may cause cataclysmic degradation of ACh leading to cognitive effects in AD patients such as memory impairment. Drugs such as donepezil, rivastigmine, and galantamine, which reversibly inhibit AChE by forming hydrolyzable carbamylated compounds with it, are widely used for symptomatic alleviation of AD [10–15]. Unfortunately, the cognitive benefit that they confer lasts only for ~ 2 years. Their actions on the peripheral cholinergic system produce side effects such as gastrointestinal disturbances, convulsions, nausea, vomiting, bradycardia, and muscle weakness, further limiting their efficacy [16]. Despite these disadvantages, a small section of the people treated with these drugs experience cognitive improvement [17], and a vast majority of people experience a delay of cognitive decline by 6–9 months [1].

Cholinesterase inhibitors increase the synaptic residence time of ACh and enhance postsynaptic cholinergic signaling [2, 18]. The exact mechanism by which this enhanced signaling translates into improved cognitive and behavioral effects remains undiscovered. Characterization of this mechanism may help us discover drugs that modulate the dementia component of AD more effectively. Detailed studies that dissect the nature of AChE inhibition and describe the influence of ACh receptor binding, ChAT activity, and enzyme localization on AChE activity are required for this characterization. Monitoring behavioral and locomotor responses to drug compounds in an appropriate model system, and studying the docking of active components of these drug compounds on AChE will provide insights into the nature of AChE inhibition.

The structure of human AChE has been characterized using chemical and kinetic studies. The active site of the enzyme contains two subsites. The breakdown of ACh into acetate and choline is catalyzed within the esteratic subsite [19]. This subsite contains the catalytic triad of three amino acids, namely, serine (Ser203), histidine (His447), and glutamate (Glu334) [20, 21]. The anionic subsite is a choline-binding pocket and interacts with the charged quaternary amine of ACh, cationic substrates, and inhibitors [19]. Apart from these, AChE also contains a distinct ‘peripheral’ anionic site at the active site entrance. This site has been implicated in substrate inhibition and allosteric regulation of ACh hydrolysis at the esteratic subsite [22].

Zebrafish (*Danio rerio*) is primarily used as a genetic model system for studying developmental and disease processes. They have biochemical and behavioral responses comparable to mammalian systems, making them suitable for drug testing. They exhibit comparable brain macro-organization, cellular morphology, neuromediator systems, and sensitivity to several classes of neurotropic drugs [23]. Localization of cholinoceptive (i.e. AChE-immunoreactive) neurons, cholinergic (i.e. ChAT-immunoreactive) neurons and AChE activity are well characterized in zebrafish [24]. AChE is the solitary cholinesterase in zebrafish [25]. AChE expression is initially found in 4 hours post-fertilization (hpf) embryos, and increases by 210-folds in 144 hpf larvae [26]. The 16 hpf embryos exhibit abrupt movements, reflecting spontaneous ACh release at the developing synaptic junctions. AChE is expressed in somites and several bilateral clusters in the presumptive brain at this stage. 21 hpf larvae become sensitive to touch stimulus and exhibit uncoordinated movements less frequently [26]. 27 hpf larvae show coordinated escape movements induced by tactile stimuli [27]. AChE localizes as large clusters in the epiphysis, forebrain, midbrain, hindbrain, and the seven rhombomeres at this stage. The 168 hpf (free-swimming) larvae exhibit the fully mature pattern of AChE activity [28].

*Convolvulus pluricaulis* (CP) is a perennial herb that has been previously studied for its anti-amnesiac and anxiolytic properties in rodents [29–32]. Aqueous CP extract has shown significant AChE inhibition in the cortex and hippocampus of male Wistar rats with scopolamine-induced cognitive impairment [30]. Two active components of CP, namely, scopoletin and scopolin, have significantly reduced scopolamine-induced amnesia in a dose-dependent manner in mice [31]. CP, in combination with rivastigmine, has inhibited aluminium-induced elevation of AChE activity in rats [32].

In this study, we studied the inhibitory mechanism of CP in zebrafish with scopolamine-induced cognitive impairment using biochemical assays, behavioral tests, and bioinformatics methods. CP showed higher avoidance response retention than isoxazole (positive control for AChE inhibition) in adult zebrafish. It exhibited inhibitory activity in the same regions as that of isoxazole in 168 hpf larvae and a concentration-dependent increase in this activity when used in combination with scopolamine. Two constituents of CP (scopoletin and kaempferol) were bound by active as well as allosteric sites of human AChE. Overall, our study proposes further investigations of CP as a modulator of cognitive brain function.

## Results and discussion

### Optimization of treatment concentrations

**Fig. 1:**
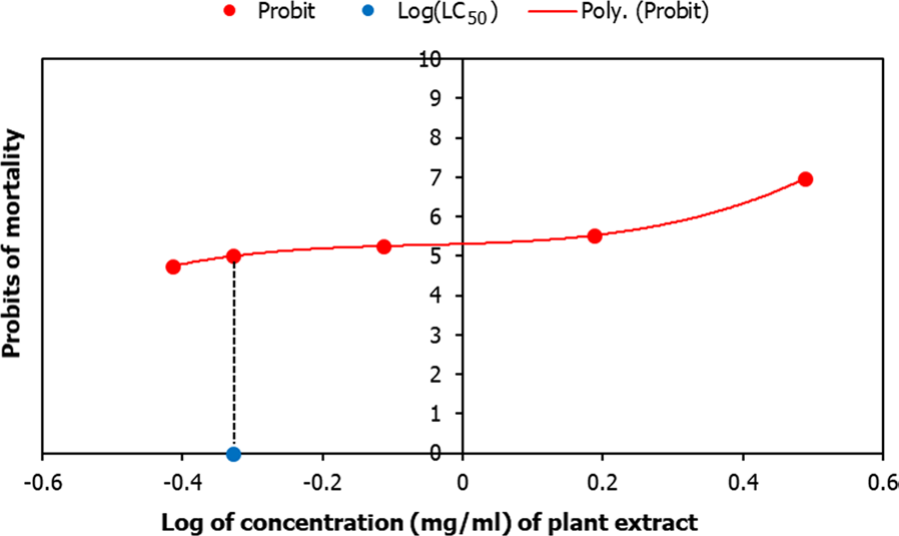
Lethal concentration (LC_50_) of *Convolvulus pluricaulis*. The graph depicts the relationship between probit mortality and the concentration of *Convolvulus pluricaulis* (CP) (depicted as solid circles). The binomial response (death/no death) of 24 hpf embryos exposed to the plant extract was recorded over 48 h. The solid line represents the third-order polynomial equation that modeled the responses, i.e. y = 8.54x^3^ + 1.61x^2^ + 0.55x + 5.31, where y is 5.00 probits. LC_50_ of 0.4708 ± 0.089 mg/ml was obtained by calculating the inverse log value of x, and standard error as (log LC_84_ − log LC_16_)/√2N

The binomial response (death/no death) of 24 hpf embryos to *Convolvulus pluricaulis* (CP) was recorded over 48 h, and the concentration-probits curve was plotted to determine lethal concentration (LC_50_) [33]. For CP, LC_50_ was determined to be 0.4708 ± 0.089 mg/mL (Fig. 1). Scopolamine (SCOP), an anti-cholinergic ligand that prevents the binding of ACh to its receptor [34], was used to induce cognitive impairment in zebrafish; this is a well-established pharmacological model of cognitive impairment. SCOP has been shown to impair both retention of learned response and acquisition of passive avoidance response in zebrafish; these cognitive deficits were rescued by the AChE inhibitor physostigmine [35]. Isoxazole (ISOX) was used as the positive control for AChE inhibition [36]. Several ISOX derivatives have exhibited inhibitory activity in vitro against AChE isolated from electric eel, rat brain, and human serum [37]. Molecular docking studies with AChE extracted from electric eel [38] and their ability to rescue scopolamine-induced amnesia in mice [39] further ascertained the utility of these compounds as AChE inhibitors. Concentrations of SCOP and ISOX were determined for 25 larvae (i.e. 40 mg of tissue), based on an estimated 200 µM of SCOP and 31.2 mM ISOX for fishes weighing ~ 1.2 g [35, 40, 41]. The least toxic concentrations of the compounds were chosen for the treatment of 25 larvae, namely 6.68 μM for SCOP, 1.04 mM of ISOX, and 0.38 mg/mL for CP. The larvae were treated with the AChE inhibitor one hour before SCOP treatment.

### The activity of *Convolvulus pluricaulis* in zebrafish larvae and adults

**Fig. 2:**
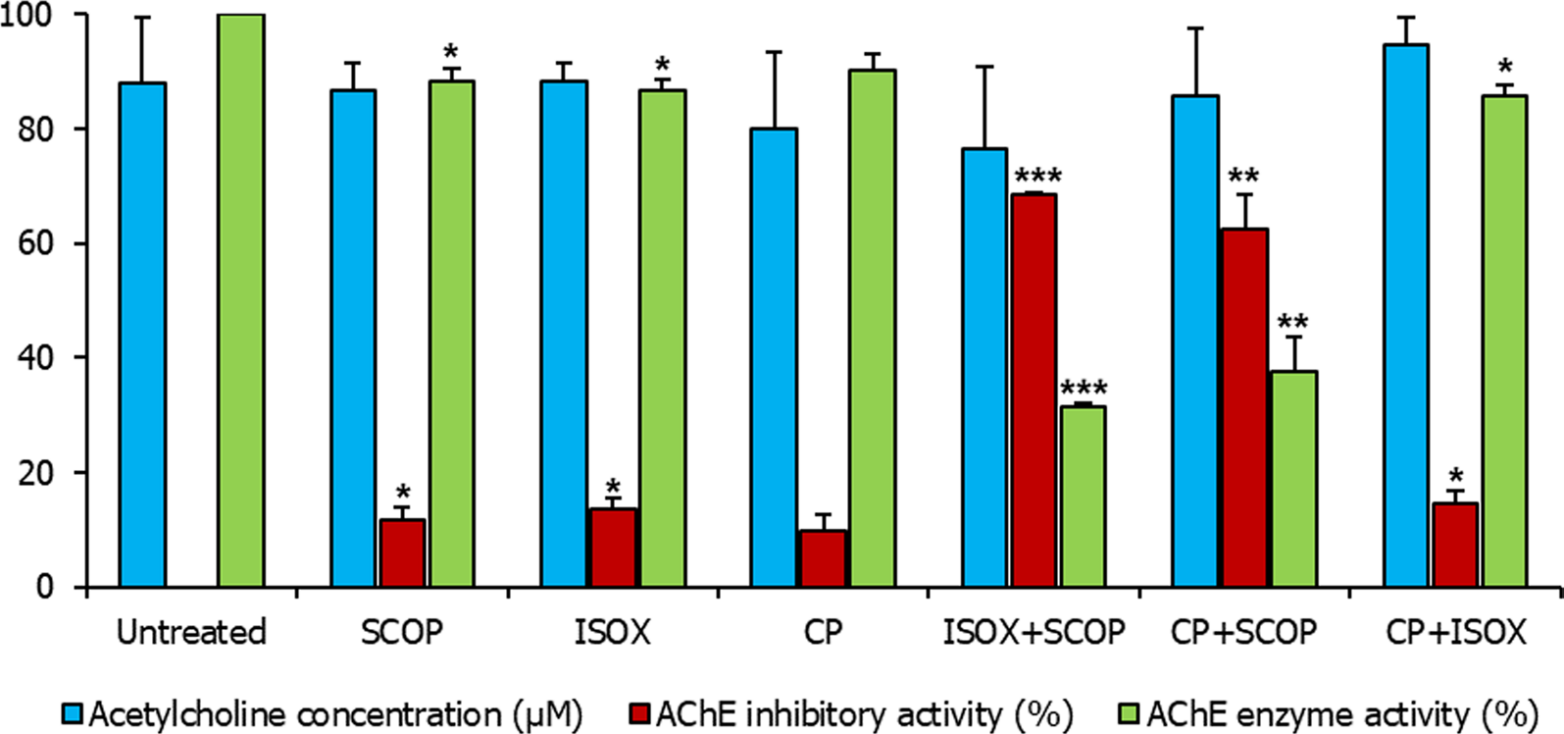
Effect of test compound(s) on acetylcholinesterase activity and acetylcholine levels. Acetylcholine (ACh) levels (blue bars), acetylcholinesterase (AChE) inhibitory activity (red bars), and acetylcholinesterase enzyme activity (green bars) in 168 hpf larvae unexposed to any test compound (i.e. ‘untreated’), treated with scopolamine (SCOP), isoxazole (ISOX), *Convolvulus pluricaulis* (CP) and their combinations (ISOX + SCOP, CP + SCOP, and CP + ISOX) have been shown. Both the ACh level (µM) and AChE activity (%) have been expressed as mean ± standard deviation. Three independent experiments were conducted to determine ACh level and AChE activity, and in each of these experiments, three sets of 15–25 embryos were treated with the least toxic concentrations of SCOP (6.68 μM), ISOX (1.04 mM), and CP (0.38 mg/ml) and their combinations. The larvae were treated with the AChE inhibitor (i.e. ISOX or CP) one hour before treatment with SCOP. *, **, and *** indicate p-value < 0.5, < 0.01 and < 0.001 of two-tailed unpaired t-test for untreated versus test groups respectively

**Fig. 3:**
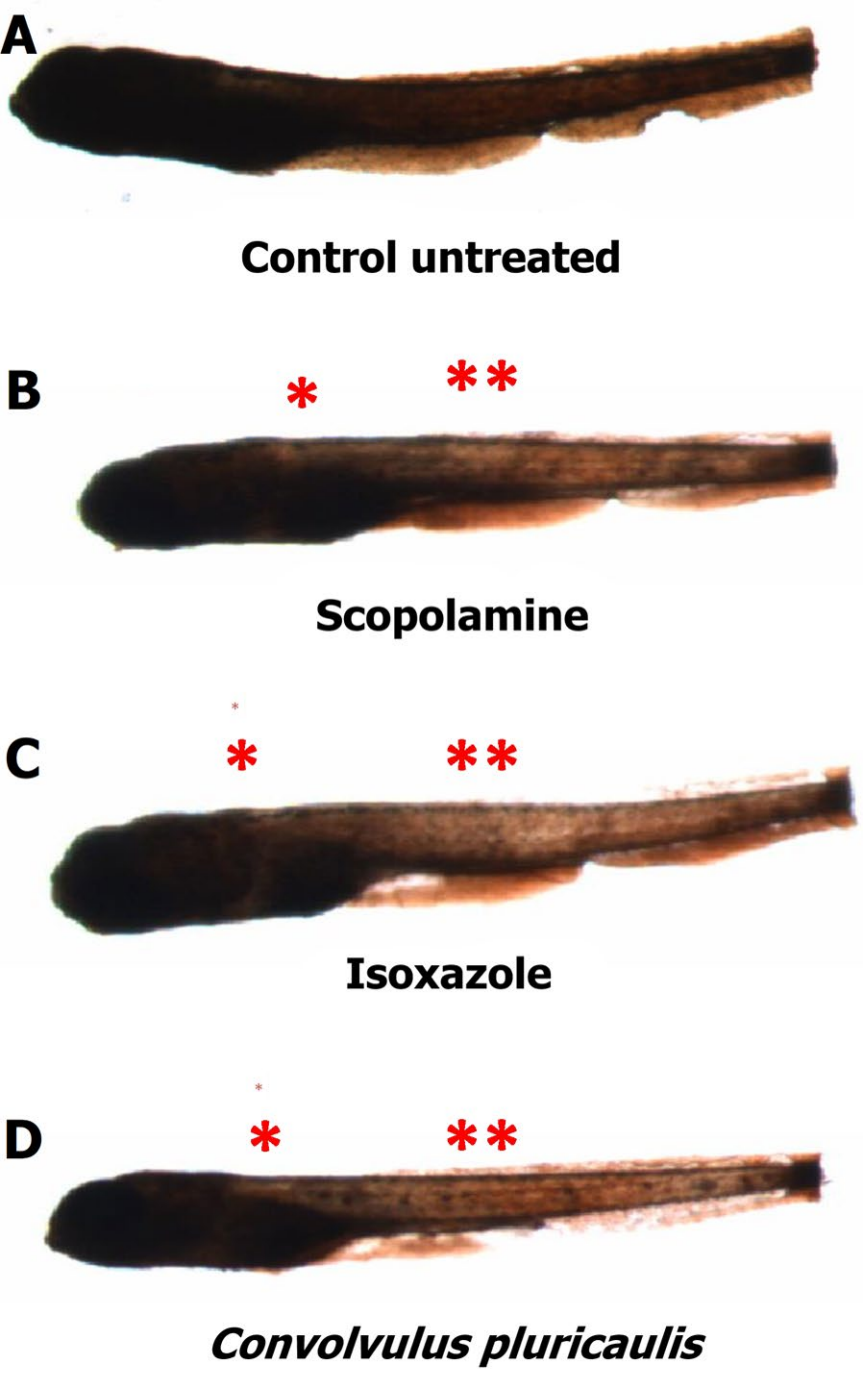
Localization of acetylcholinesterase activity in larvae treated with test compounds. The AChE activity in **A** untreated 168 hpf larvae and larvae treated with **B** scopolamine, **C** isoxazole, and **D** *Convolvulus pluricaulis* was visualized using Karnovsky–Roots staining. The intensity of the brown stain is indicative of the level of AChE activity. * indicates AChE inhibition in myelencephalon and ** indicates AChE inhibition in somites containing sensory interneurons and motor units

The effect of CP on AChE activity and ACh levels in 168 hpf zebrafish larvae treated with SCOP was studied using Ellman’s assay and the hydroxylamine method (Fig. 2). We also employed the Karnovsky staining method for qualitative analysis of AChE activity in 168 hpf larvae [42]. The ACh level in untreated control larvae was found to be 87.86 ± 1.61 µM. Karnovsky staining revealed a mature pattern of AChE activity in these larvae (Fig. 3A). On treatment with the AChE inhibitor, ISOX, the level only slightly increased to 88.33 ± 3.12 µM, and the inhibitory activity was found to be 13.48% ± 1.92 (p-value = 1.97E−02). Clearance of the Karnovsky stain was interpreted as AChE inhibition. Visual inspection revealed that ISOX showed AChE inhibition in the myelencephalon and the somites containing sensory interneurons and motor neurons (Fig. 3C). With CP, the inhibitory level was 9.76% ± 2.94 and the ACh level was at 79.83 ± 13.44 µM, which was not significantly different from the levels in untreated larvae. However, CP also showed stain clearance in the myelencephalon and the somites (Fig. 3D). Overall, the pattern of clearance shown by ISOX and CP in these regions seemed to be similar (Fig. 3C, D), indicating that CP may exert an inhibitory effect comparable to that of the positive control.

**Fig. 4:**
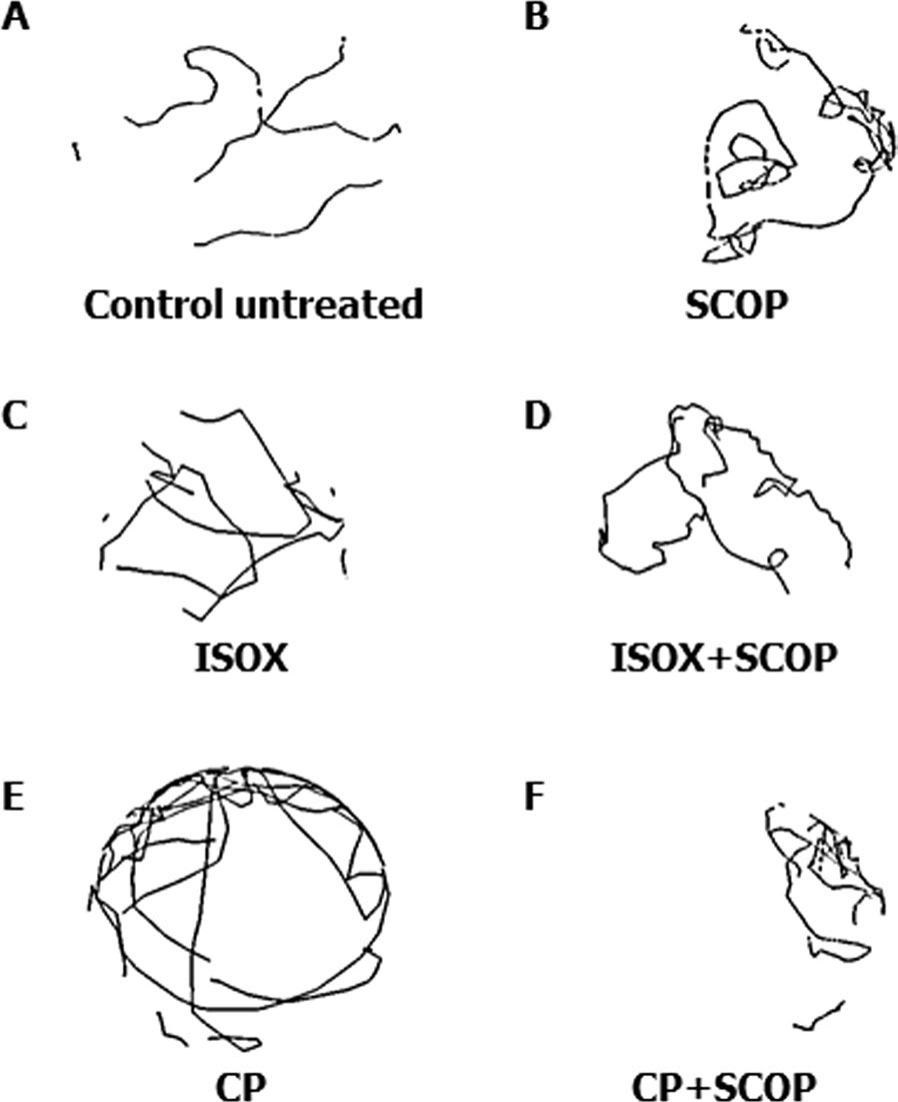
Swimming patterns of larvae and adults in response to test compounds. The locomotion tracking plots (or ‘traces’) extracted from wrMTrck for **A** untreated, **B** scopolamine (SCOP)-treated, **C** isoxazole (ISOX)-treated, **D** ISOX + SCOP-treated, **E** *Convolvulus pluricaulis* (CP)-treated, and **F** CP + SCOP-treated larvae. The 168 hpf larvae were treated with the least toxic concentrations of SCOP (6.68 μM), ISOX (1.04 mM), and CP (0.38 mg/mL) and their combinations. Note that each larva is represented by a single trace. Three independent experiments were conducted with each of the treatment conditions to determine the locomotion patterns of the larvae. Three minutes long videos (2 min for acclimatization and 1 min for test response) of 3 larvae (per Petri dish placed on a light source) were shot in a dark chamber

Using the wrMTrck software optimized for zebrafish, we inspected the locomotor patterns of the larvae treated with the different test compounds (Fig. 4) [43, 44]. Visual inspection revealed that the movement patterns of larvae treated with individual compounds such as ISOX, SCOP, and CP were distinguishable from the paths of untreated control larvae (Fig. 4A–C, E). The overlapping paths of the treated larvae compared to the non-overlapping paths of control larvae indicated inadequate sensory reception and motor control in the treated larvae. We had found the inhibitory activity of CP to be localized to the myelencephalon and the somites containing small sensory interneurons and large motor neurons (Fig. 3). Myelencephalon regulates the anti-predatory escape response in zebrafish larvae, the stimuli for which are conveyed by the hair cells of the lateral line system innervated by large motor neurons [45]. This C-start startle escape response regulated by Mauthner cells in the zebrafish hindbrain is modulated by a form of non-associative memory called habituation [46]. As expected, we found abnormal body bends and deregulated coordination of motor responses in CP-treated larvae (Fig. 4E). This also shows that CP can enter and act on the lateral line system of zebrafish larvae, producing abnormal locomotor responses, and thereby limiting its value as a therapeutic candidate for cognitive impairment in AD. However, further studies are required to examine these motor responses in detail.

**Fig. 5:**
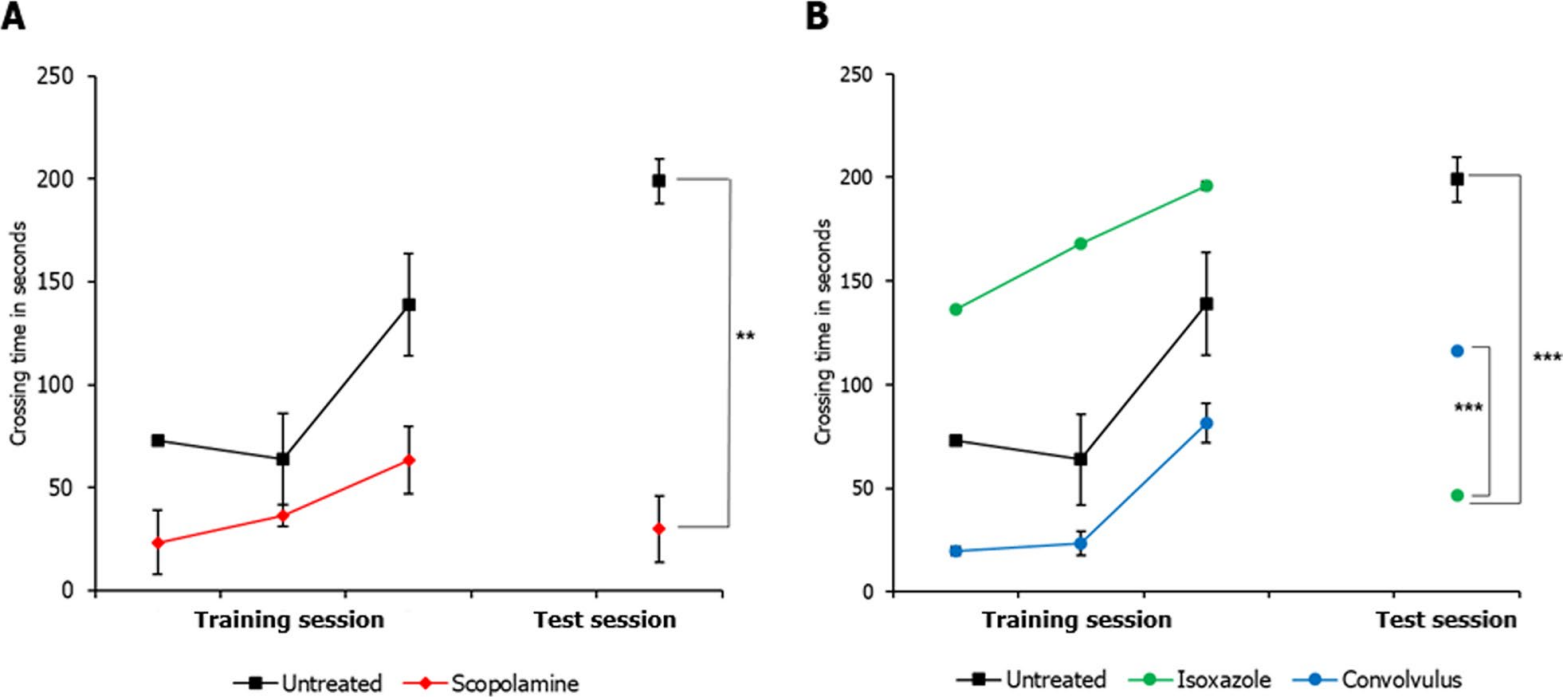
Effect of test compounds on the acquisition of avoidance response. Comparison of the crossing times of **A** control untreated and scopolamine (SCOP)-treated adults and **B** adults treated with isoxazole (ISOX) and *Convolvulus pluricaulis* (CP). The solid circles and squares represent the crossing times measured in the three training sessions and one test session, which was conducted 2 h after the training sessions. These crossing times have been expressed as mean ± standard deviation. Three independent experiments were conducted to determine the effects of SCOP, ISOX, and CP on the fishes, and in each of these experiments, at least 3–5 adult fishes were treated with the least toxic concentrations of SCOP (200 µM), ISOX (31.2 mM) and CP (11.4 mg/ml). The fishes were treated with the AChE inhibitor (i.e. ISOX or CP) one hour before treatment with SCOP. *, **, and *** indicate p-value < 0.5, < 0.01 and < 0.001 of two-tailed unpaired t-test for untreated versus test groups (in most cases, unless indicated otherwise) respectively

A passive avoidance response test was used to test the acquisition of avoidance response in adult zebrafish. As expected, SCOP-treated adults failed to acquire the response (p-value = 8.2E−03) (Fig. 5A). ISOX-treated fishes showed greater acquisition of response compared with untreated fishes during the training session (p-value = 3.55E−02) but did not retain the memory in the test session (Fig. 5B). Although CP-treated fishes showed lower acquisition of response than control during the training session, they showed higher retention of memory than isoxazole in the test session (p-value < 1E−04), indicating that CP may be superior to the positive control in memory retention (Fig. 5B).

**Fig. 6:**
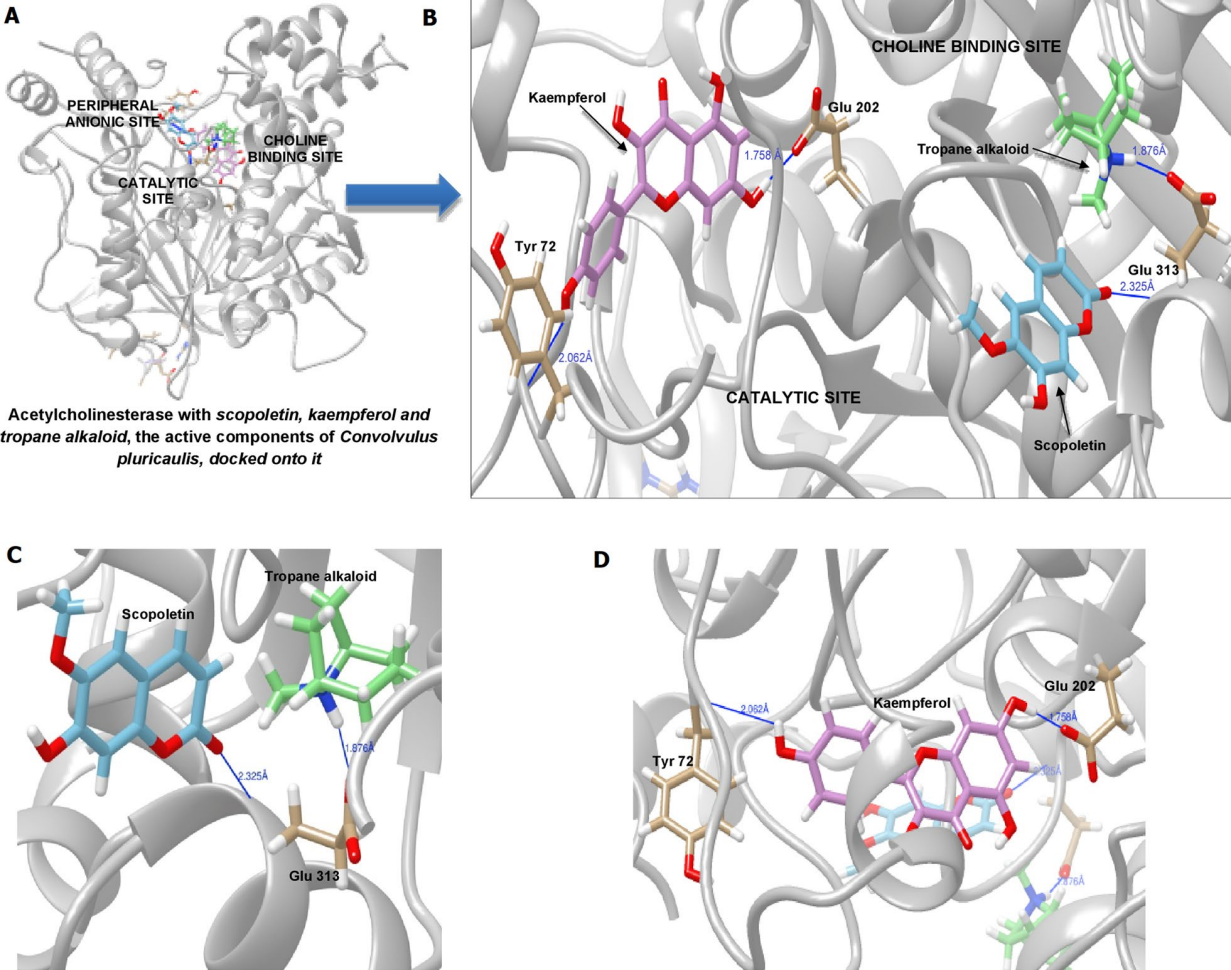
Binding of Convolvulus active components to acetylcholinesterase. **A**, **B** Various pockets on acetylcholinesterase to which the active components, namely, scopoletin (and its central tropane alkaloid structure) and kaempferol, were bound, **C** Scopoletin and tropane alkaloid at Glu313, **D** Kaempferol binding at Tyr72 and Glu202

We studied the binding of the two active components of CP—scopoletin, and kaempferol—on human AChE using molecular docking (SwissDock [47], see Sect. 4). The crystal structure of human AChE was extracted from PDB ID: 4PQE (Fig. 6 and Table 1). In the absence of its substrate ACh, AChE was bound by the positive control ISOX at its catalytic site—specifically at Ser203 [20, 21]—with a binding energy of − 14.03. When AChE was bound by ACh at its primary binding site Hsd405 in the choline-binding pocket, ISOX was found at Ser203 with a lower binding energy of − 14.11. Scopoletin (an active component of CP) was found to bind to Glu313 (via tropane, the central structure of scopoletin), the site bound by SCOP (Fig. 6B, C and Table 1). Scopoletin also establishes contact with His447 of the catalytic triad [20, 21] and Glu202 in the peripheral anionic site (Table 1). Kaempferol was found to bind to Glu202 and Tyr72 in the peripheral anionic site (Fig. 6B, D and Table 1).

**Table 1:** Details on critical interactions of acetylcholinesterase with isoxazole, scopolamine and active components of *Convolvulus pluricaulis*, namely, scopoletin and kaempferol, and the central structure of scopoletin (tropane alkaloid)

Target	Ligand	Hydrogen bond forming residues	Binding energy
AChE	Acetylcholine	Hsd 405	− 13.5878
AChE + acetylcholine	Acetylcholine	Phe 295	− 12.2174
AChE	Isoxazole	Ser 203	− 14.0329
AChE + scopolamine	Isoxazole	Phe 295	− 12.7298
AChE + scopolamine	Isoxazole	Hsd 405	− 12.9197
AChE + acetylcholine	Isoxazole	Ser 203	− 14.1166
AChE + acetylcholine	Isoxazole	Phe 295	− 12.7298
AChE + acetylcholine + scopolamine	Isoxazole	Ser 203	− 13.9634
AChE	Scopolamine	Gly 234, Thr 238	223.762
AChE + isoxazole	Scopolamine	Lys 53	226.227
AChE + acetylcholine	Scopolamine	Glu 313	223.469
AChE + acetylcholine + acetylcholine	Scopolamine	Glu 313	219.043
AChE + acetylcholine + isoxazole	Scopolamine	Glu 313	222.987
AChE + acetylcholine + acetylcholine	Atropine	Glu 313	− 4.475
AChE	Galantamine	Glu 313	0.951452
AChE	Galantamine	Lys 53, Glu 185	6.7258
AChE + acetylcholine + acetylcholine + scopolamine	Isoxazole	Ser 203	− 14.1095
AChE	Scopoletin	Glu 313	23.1182
AChE	Scopoletin	His 447 (HSP)	17.3092
AChE	Scopoletin	Glu 202	17.3475
AChE	Tropane alkaloids	Glu 313	− 16.981
AChE	Kaempferol	Glu 202, Tyr 72	10.6597

The binding mode of scopoletin on AChE—specifically, its ability to occupy the ACh-binding anionic subsite (in the catalytic center)—suggests that it may act as a competitive inhibitor of ACh. This observation is supported by previous studies that demonstrated its AChE inhibitory activity in vitro [31, 48] and ability to increase extracellular ACh concentration in rat brains to a level comparable to that of galantamine [48], a compound often used as a positive control for AChE inhibition. However, scopoletin may enhance ACh levels via mechanisms other than AChE inhibition, such as agonistic activity on nicotinic ACh receptors, which increases ACh release from synaptosomes [49]. Although our results indicate that competitive inhibition of AChE by scopoletin may increase ACh levels, further investigations may be necessary to elucidate the exact mechanisms. Besides, the disease-modifying effects of scopoletin possibly mediated by its additional interactions with the peripheral anionic and esteratic sites of the enzyme also need to be examined further. Unlike scopoletin, kaempferol (a flavonoid) may allosterically modulate the conformation of the catalytic triad or block ACh entry into the active site of the enzyme by binding to the peripheral anionic site as a non-competitive inhibitor, a feature exhibited by flavonoids [50]. Kaempferol has been shown to strongly inhibit AChE in a previous study [51]. Overall, in contrast with the positive control ISOX that only acts on the catalytic triad, the active components of CP may bind to the choline-binding pocket and the peripheral anionic site and mediate both competitive and non-competitive modes of inhibition.

It must be noted that our docking analysis was limited to only two phytoconstituents of CP (scopoletin and kaempferol) that have shown anti-AChE activity in separate studies. A more comprehensive analysis including alkaloid, anthocyanin, triterpenoid and phytosterol components of CP [52] should be conducted to fully characterize the AChE inhibitory activity of CP.

### The inhibitory activity of scopolamine in zebrafish larvae

An inhibitory activity of 11.68% ± 2.28 was noted in SCOP-treated larvae compared to untreated larvae (p-value = 3.61E−02); the ACh level was 86.54 ± 4.90 µM (Fig. 2). This was supported by qualitative analysis for AChE activity as well (Fig. 3B). To the best of our knowledge, our study is the first to report the AChE inhibitory activity of SCOP in zebrafish. However, several studies in the past have demonstrated the AChE inhibitory activity of SCOP or its analogs in other animal models. Cholinergic ligands such as atropine, hyoscyamine, and gallamine have been known to show inhibitory activity on AChE [53, 54]. For example, atropine—which is structurally similar to SCOP—inhibits AChE in the presence of low concentrations of acetylthiocholine iodide (K_i_ = 4 × 10^–3^) [53]. It was shown in guinea pigs that SCOP administered at a concentration of 1.94 µg/h for 6 days inhibited red blood cell AChE by 18.7% ± 3.7 and plasma cholinesterase by 44.1% ± 3.1 [54]. SCOP showed competitive AChE inhibition at a concentration of 0.25 × 10^–2^ M and mixed type AChE inhibition at a concentration of 0.5 × 10^–2^ M in synaptosomal fractions isolated from rat brain [55].

**Fig. 7:**
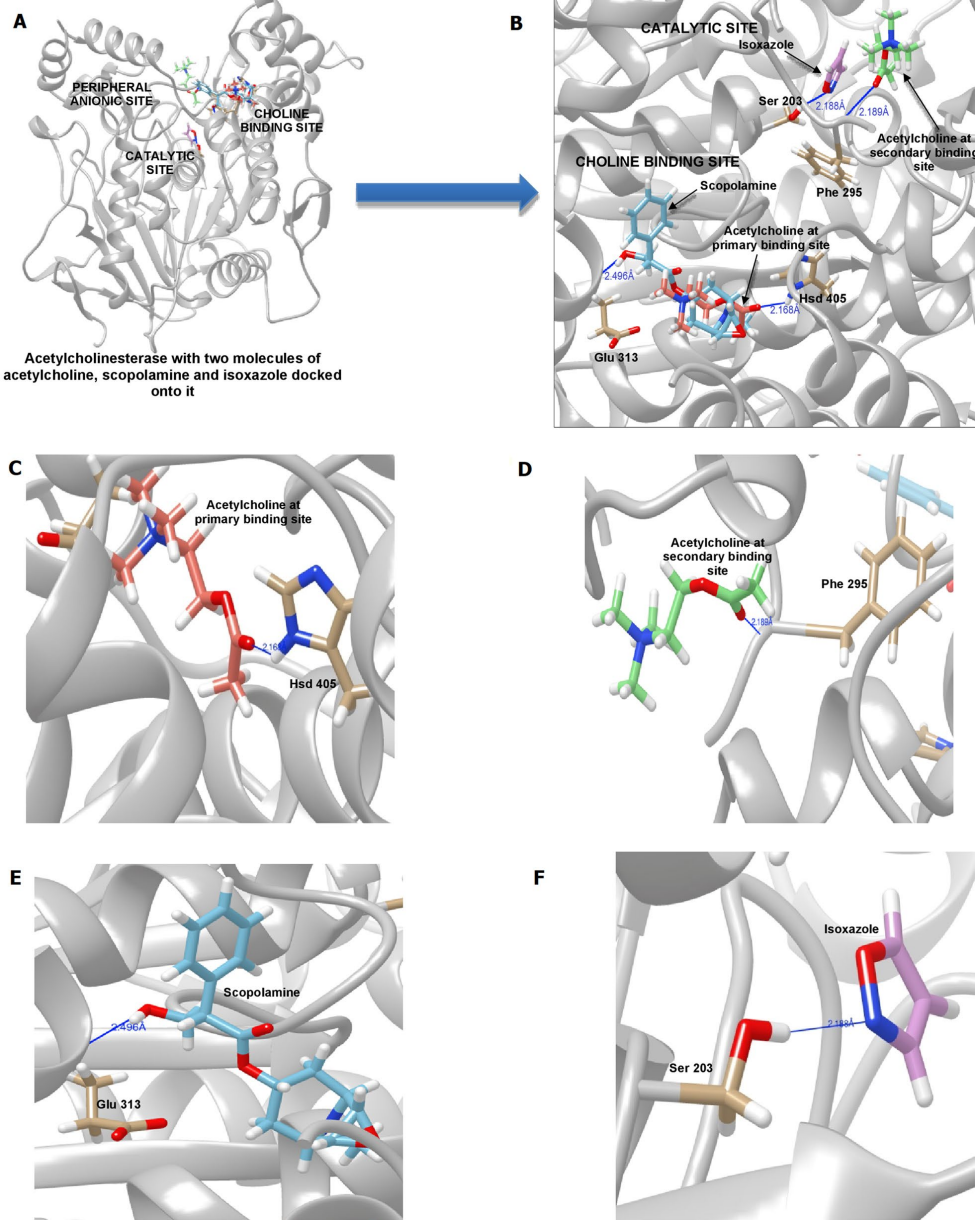
Binding of isoxazole and scopolamine to acetylcholinesterase. **A**, **B** Various pockets on acetylcholinesterase to which its substrate acetylcholine, isoxazole and scopolamine were bound, **C** Acetylcholine at Hsd405, the primary binding site, in the choline-binding pocket, **D** Acetylcholine at Phe295, the secondary binding site, near the catalytic site, **E** Scopolamine at Glu313 in the choline-binding pocket, **F** Isoxazole binding Ser203, a catalytic triad residue

Using molecular docking, we found that, in the absence of ACh, AChE was bound by SCOP at Gly234 and Thr238 in the peripheral anionic site with a binding energy of 223.762, the former residue being the one that mediates the inhibitory effect of galantamine, an AChE inhibitor [56]. In the presence of a single molecule of ACh at Hsd405, SCOP established hydrogen bonds with Glu313, the same site with which atropine interacts at a binding energy of − 4.475. Atropine is structurally identical to SCOP, except for a highly reactive epoxide group that the latter bears. These epoxides are highly reactive (compared to simple ethers) because of their ring strain. Nucleophilic attack of the electrophilic C in the C–O bond causes the ring to open. The energy of a simple epoxide, ethylene oxide, changes from − 8.95 to 181.04, after protonation [57]. In the presence of ACh molecules at the primary (Hsd405) and secondary (Phe295) sites (Fig. 7C, D), the binding energy of SCOP and AChE is 219.043 (Fig. 7B, E). SCOP may be primed to be bound by AChE after protonation of the epoxide group. This hypothesis should be validated through a dynamic simulation of the binding of SCOP to AChE. Nevertheless, our data indicate that the AChE inhibitory activity of SCOP may be mediated via its binding to the two ACh-binding sites, the anionic subsite of the catalytic center and the peripheral anionic site.

### The combinatorial effect of *Convolvulus pluricaulis* and scopolamine

**Fig. 8:**
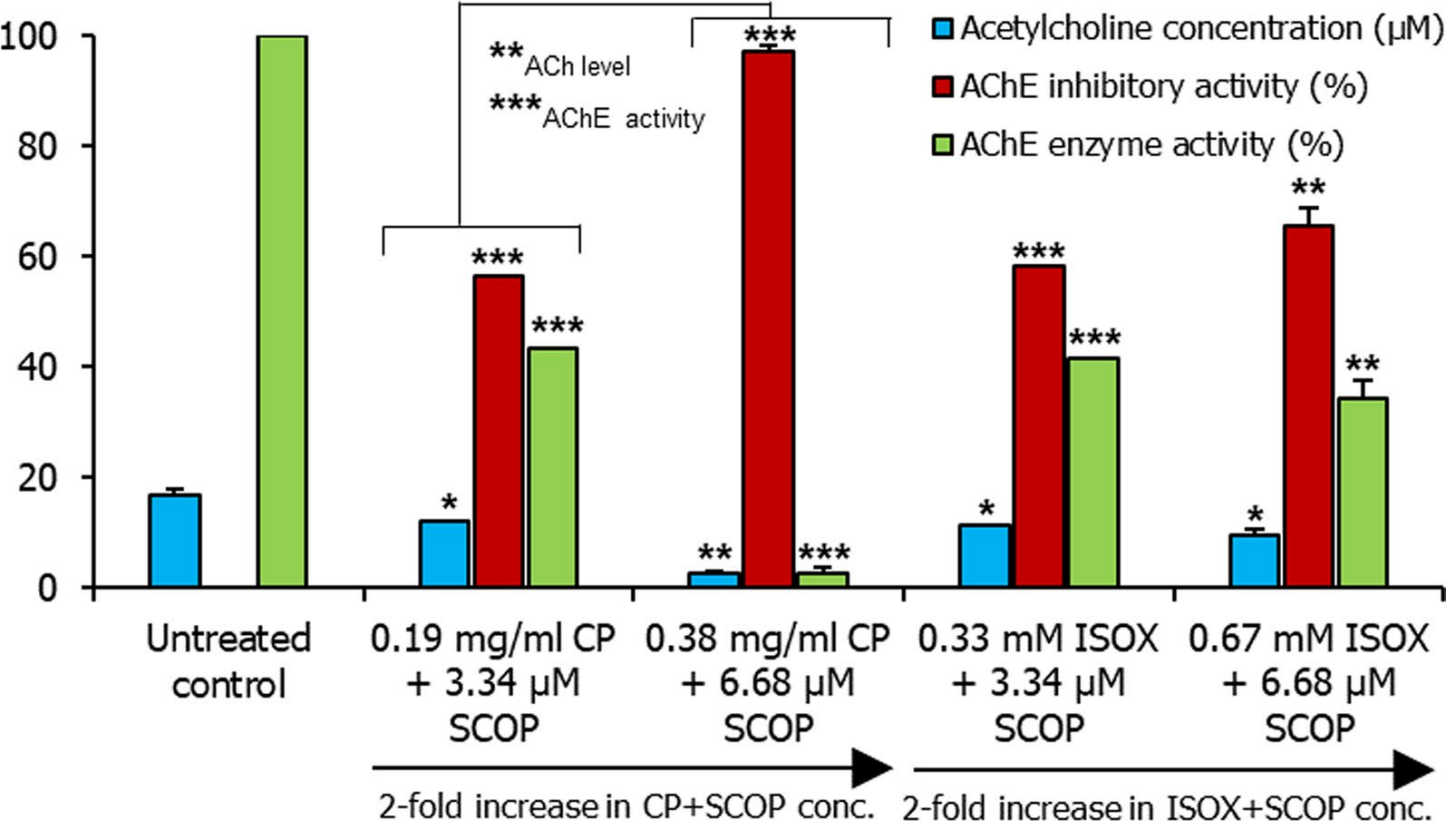
Concentration-dependent effect of CP + SCOP and ISOX + SCOP on acetylcholinesterase activity and acetylcholine levels. Acetylcholine (ACh) levels (blue bars), acetylcholinesterase (AChE) inhibitory activity (red bars), and acetylcholinesterase enzyme activity (green bars) in 168 hpf larvae unexposed to any test compound (i.e. ‘untreated’) and treated with the combinations of scopolamine (SCOP) with *Convolvulus pluricaulis* (CP) and isoxazole (ISOX), i.e. CP + SCOP and ISOX + SCOP, in varying concentrations have been shown. Two concentrations were tested for each of these combinations. For CP + SCOP, these were 0.19 mg/ml of CP + 3.34 μM of SCOP and 0.38 mg/mL of CP + 6.68 μM of SCOP. For ISOX + SCOP, these were 0.52 mM of ISOX + 3.34 μM of SCOP and 1.04 mM of ISOX + 6.68 μM of SCOP. Both the ACh level (µM) and AChE activity (%) have been expressed as mean ± standard deviation. Three independent experiments were conducted to determine ACh level and AChE activity, and in each of these experiments, three sets of 15–25 embryos were treated with the said concentrations. The larvae were treated with the AChE inhibitor (i.e. ISOX or CP) 1 h before treatment with SCOP. *, **, and *** indicate p-value < 0.5, < 0.01 and < 0.001 of two-tailed unpaired t-test for untreated versus test groups respectively

A combination of ISOX and SCOP (ISOX + SCOP) increased the inhibitory activity of AChE to 68.45% ± 0.5 (p-value < 1E−04) compared to either of their inhibitory activities in isolation (Fig. 2). Similarly, a combination of CP and SCOP (CP + SCOP) increased the inhibitory activity to 62.5% ± 6.065 (p-value = 9.3E−03) compared to their inhibitory activities in isolation (Fig. 2). To investigate the combinatorial effect of ISOX + SCOP and CP + SCOP further, we increased the concentration of these compounds in tandem (Fig. 8). The increase in inhibitory activity with increasing concentrations was significant in CP + SCOP (p-value < 1E−02), but not in ISOX + SCOP (Fig. 8). Hence, CP, and not ISOX, seemed to significantly enhance AChE inhibition in a synergistic and concentration-dependent manner with SCOP. Contrary to an expected increase in ACh levels in this scenario, a significant drop in ACh level (p-value < 1E−03) was noted with CP + SCOP (Fig. 8). The drop in ACh levels in the different concentrations of ISOX + SCOP and CP + SCOP was significant compared to untreated control (Fig. 8). SCOP, which is synergistically inhibiting AChE together with CP, may be rendered unavailable for binding with the ACh receptor. In this scenario, the ACh pool enhanced as a result of the inhibitory activity mediated by CP + SCOP may be rapidly depleted through its binding with the ACh receptor. SCOP has been previously shown to decrease cerebral ACh levels by 31% at a concentration of 0.63 mg/kg [58]. It has also been noted that SCOP is more potent than atropine in reducing ACh levels [58]. Further experiments are necessary to test our speculations.

**Fig. 9:**
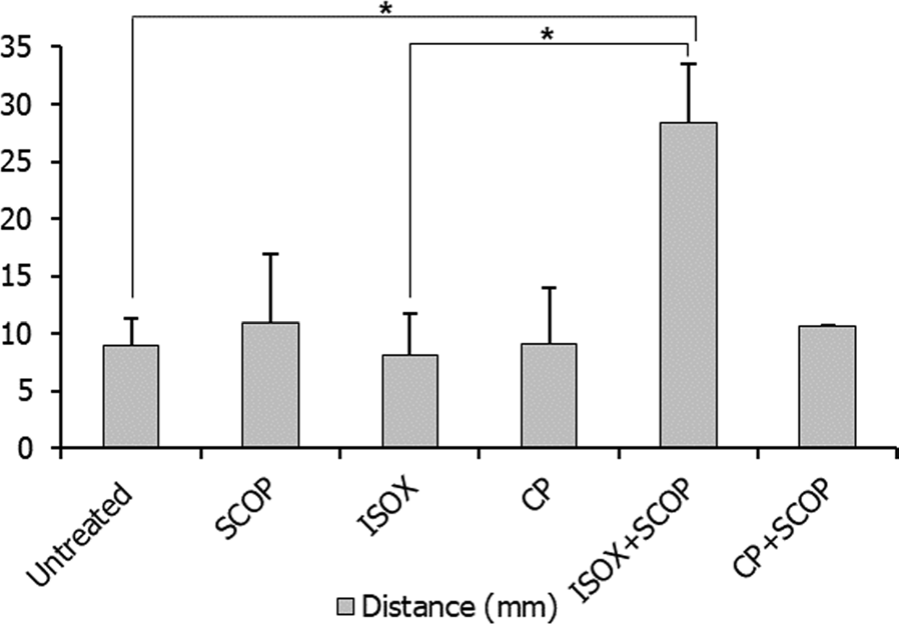
Distance covered by the larvae treated by the test compounds. Histograms of the distances covered by untreated, scopolamine (SCOP)-treated, isoxazole (ISOX)-treated, *Convolvulus pluricaulis* (CP)-treated, ISOX + SCOP-treated, and CP + SCOP-treated larvae. Three independent experiments were conducted with each of the treatment conditions. *, **, and *** indicate p-value < 0.5, < 0.01 and < 0.001 of two-tailed unpaired t-test for untreated versus test groups (unless indicated otherwise) respectively

We studied the locomotor patterns of larvae treated with ISOX + SCOP and CP + SCOP. Larvae treated with ISOX + SCOP covered increased distances in comparison with ISOX- treated larvae (p-value = 8.25E−02) (Fig. 9). Larvae treated with SCOP had executed a whirling motion in an uncoordinated direction with high speed over a short distance (Fig. 4B). This locomotor type, known as corkscrew swimming, is commonly observed as part of a seizure phenotype [59]. This would have arisen from the inhibitory activity of SCOP in sensory interneurons and motor units, rendering cholinergic transmission uncoordinated at the cholinergic synapse. In ISOX + SCOP and CP + SCOP, this whirling motion characteristic of SCOP added up to the locomotor repertoire of the treated larvae (Fig. 4D, F). Larvae treated with ISOX + SCOP (Fig. 4D) executed a zigzag motion often produced in response to alarming stimuli (chemical cue in our case) [59]. However, ISOX + SCOP-treated adults seemed to exhibit boldness, a behavior often accompanied by an increased approach towards novel objects [59]. They spent more time in the central portion of the tank than the peripheral areas compared to untreated and ISOX-treated adults (Additional file 1: Fig. S1). This could indicate that distinct behavioral repertoires characterize the different life stages of the zebrafish. Nevertheless, ISOX + SCOP and CP + SCOP disrupt motor response patterns characteristic of specific stages of life.

We studied the binding of ISOX and SCOP on AChE using molecular docking. When AChE was bound by SCOP at Glu313 in the choline-binding pocket, ISOX was bound by the enzyme at the primary and secondary ACh binding sites at a higher energy of − 12.91 to − 12.72. However, in the presence of ACh at Hsd405, SCOP was bound by the enzyme at Glu313 and ISOX at Ser203 with a lower binding energy of − 13.96. An even lower binding energy of − 14.1 was observed with AChE binding two molecules of ACh at Hsd405 and Phe295 (Fig. 7C, D and Table 1), SCOP at Glu313 and ISOX at Ser203 (Fig. 7B, F and Table 1), indicating that this configuration of ISOX + SCOP at the various subsites in the catalytic center may be responsible for its synergistic activity on the enzyme. Similar studies should also be conducted with CP + SCOP to elucidate their mechanism of inhibition.

## Conclusions

This study was undertaken to gain new mechanistic understanding into the modes of AChE inhibition of CP, known to have anti-AChE activity. CP-treated 168 hpf larvae showed a similar pattern of AChE inhibition (in the myelencephalon and somites) as that of the larvae treated with the AChE inhibitor ISOX, which was used as a positive control. Additionally, CP improved the retention of avoidance response in adult zebrafish compared with ISOX. ISOX was found to directly bind Ser203 of the catalytic triad on the human AChE. The active components of CP—scopoletin and kaempferol—were found to bind to His447 of the catalytic triad, the anionic subsite of the catalytic center, and the peripheral anionic site. Unexpectedly, SCOP, which was used in our study to induce cognitive impairment in zebrafish, showed AChE inhibition in 168 hpf larvae, possibly mediated via the anionic subsite of the catalytic center and the peripheral anionic site, as indicated by docking studies with human AChE. Interestingly, CP + SCOP significantly increased AChE inhibition and depleted ACh levels compared with untreated larvae, a pattern that was also observed albeit in a statistically non-significant way in ISOX + SCOP. Abnormal motor responses were observed individually with ISOX and CP, and in their combinations with SCOP, indicative of undesirable effects on the peripheral cholinergic system. Our study proposes the examination of CP, SCOP, and CP + SCOP as potential AChE inhibitors for their ability to modulate cognitive deficits in Alzheimer’s disease.

## Materials and methods

### Collection of zebrafish embryos

Adult wild-type zebra fish were maintained in tanks as per standard conditions [60]. Spawning was set up every 5 to 6 days in large troughs, usually 2 to 3 h after feeding. The natural mating ratio of zebrafish is 1 female:2 males. Females and males were housed for spawning either in this ratio or in equal numbers. Fertilization occurs in the early hours of the morning. Eggs were collected at the 4 hours post-fertilization (hpf) stage and transferred to large Petri plates containing the E3 medium (1–5 mM NaCl, 0.17 mM KCl, 0.33 mM CaCl_2_, 0.33 mM MgSO_4_, 10–5% Methylene Blue). The developmental stage of the embryos was observed under a microscope [60]. Embryos showing asynchronies in development stages and abnormal development (detectable after 10 hpf) were segregated from the rest. Dead embryos were separated every 4 h. Swimming larvae at 168 hpf were used for this study.

### Preparation of plant extract

An aqueous solution of the root of *Convolvulus pluricaulis* (CP) was prepared by agitating 2 g of macerated root in 100 ml of distilled water at 150 rpm at room temperature for 24 h. The solution was clarified by filtration, frozen overnight at – 20 °C, and lyophilized for 18 h to obtain 18.5 mg of the extract.

### Determination of lethal concentration, 50% (LC_50_) of the plant extract

24 hpf embryos were treated with the plant extract in 96 well plates according to Zebrafish Embryo Toxicity Test (ZFET) Protocol Standards [61]. ZFET allows assessment of the phenotypes manifested by fish embryos on treatment with chemicals. The lethal dose for a plant preparation is the particular concentration at which half of the embryos treated with the preparation are alive. Binomial response (death/no death) of 24 hpf embryos to CP was recorded over 48 h and concentration-probits mortality curve was plotted to determine LC_50_ [33].

### Ellman’s assay

Ellman’s assay is a spectrophotometric method that quantifies AChE activity in terms of μ moles of acetylthiocholine iodide (ATCh-I) hydrolyzed per minute per mg of the plant preparation [62]. A key player in this assay is the DTNB reagent also known as Ellman’s reagent. The mechanism by which it quantifies the amount of substrate hydrolyzed is depicted below:
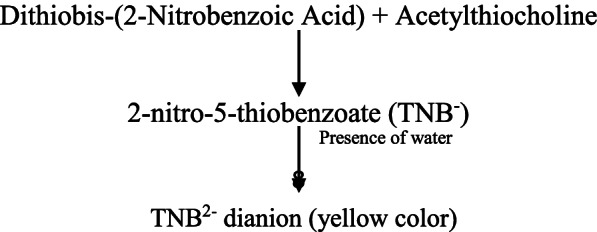


The reagents for Ellman’s assay included 0.1 M phosphate buffer saline (PBS) at pH 7.0, 0.075 M acetylthiocholine iodide (ATCh) as substrate, and 0.01 M DTNB (dithiobis-(2-nitrobenzoic acid)), prepared by dissolving 39.6 mg in 10 ml phosphate buffer (0.1 M) at pH 7.0 and adding 15 mg of sodium bicarbonate. The digestion buffer was prepared by adding 20 mM Tris/HCl at pH 7.0, 5 mM EDTA and 1% Triton X-100. Embryos were euthanized using a mixture of 1 mL of clove oil and 9 mL of absolute ethanol. 1 mL of this solution was then dissolved in 50 mL of tap water. This solution was then transferred to a Petri dish with 20 embryos to be euthanized. 15–25 embryos were suspended in 150–250 µL digestion buffer (20 mM Tris/HCl pH 7.0, 5 mM EDTA, 1% Triton X-100) and homogenized by pipetting the suspension in and out. The homogenate was centrifuged at 1500 rpm for 15 min. The supernatant diluted in 0.1 M phosphate buffer at pH 7.0 was used as the enzyme (AChE) source. A blank reaction mixture was prepared with the phosphate buffer, substrate, plant extract solutions at specific concentrations, and DTNB reagent. Test reaction mixtures were prepared with the phosphate buffer, substrate, enzyme source, plant extract solutions at specific concentrations, and DTNB reagent. Absorbance was measured at 412 nm.

### Hydroxylamine method for acetylcholine estimation

Hydroxylamine reacts in a strongly alkaline medium with the substrate (ACh) forming acetohydroxamic acid and methanol. Acidification of this mixture with HCl and the addition of Fe^3+^ ions result in a red-brown complex, ACh-acetohydroxamic product, which can be detected through colorimetry [63]. The solutions used for this assay included 2 M aqueous hydroxylamine hydrochloride, 3.5 M aqueous potassium hydroxide, conc. HCl/H_2_O (in 1:2 ratio), 0.37 M Fe^3+^ (as ferric nitrate or ferric chloride) in aqueous 0.1 M HCl and a standard aqueous solution of 4 mM. Embryos were euthanized and washed. 15–25 embryos were suspended in 150–250 µL digestion buffer and homogenized by pipetting the suspension in and out. The homogenate was centrifuged at 1500 rpm for 15 min. The supernatant diluted in 0.1 M phosphate buffer at pH 7.0 served as the source for the enzyme (AChE) and the substrate (ACh). The blank reaction mixture was prepared with the phosphate buffer and the enzyme and substrate source. Test reaction mixtures were prepared with the phosphate buffer, the enzyme and substrate source, and the plant extract solution at specific concentrations. The reaction mixture was vigorously mixed with aqueous hydroxylamine hydrochloride and aqueous potassium hydroxide in the ratio of 1:1. The rapid change in pH stops hydrolysis. The resulting mixture was then mixed for 2 min to allow conversion of ACh to acetohydroxamic acid. The pH was then changed to 1.2 by adding conc. HCl/H_2_O and aqueous ferric nitrate or ferric chloride. Absorbance was measured at 540 nm. The concentration of ACh was calculated using its molar absorption coefficient (Ɛ(Ach, 540 nm, 25 °C) = 785 M^−1^ cm^−1^).

### Karnovsky’s direct coloring thiocholine method for cholinesterases

Karnovsky’s staining method was used for the visualization of localized AChE activity [42]. The mechanism by which this staining method generates a color reaction in response to AChE activity is depicted below:

$$\begin{gathered} {\text{Acetylthiocholine }}\left( {{\text{substrate}}} \right)\mathop{\longrightarrow}\limits^{{^{{{\text{AChE source }}\left( {{\text{embryos}}} \right)}} }}{\text{Acetic acid}} + {\text{Thiocholine}} \hfill \\ {\text{Ferricyanide }}\left( {{\text{Fe}}^{{{3} + }} } \right)\mathop{\longrightarrow}\limits^{{^{{{\text{Thiocholine}}}} }}{\text{Ferrocyanide }}\left( {{\text{Fe}}^{{{2} + }} } \right) \hfill \\ {\text{Copper }}\left( {{\text{Cu}}^{{{2} + }} } \right) + {\text{Ferrocyanide}} \to {\text{Copper ferrocyanide }}\left( {\text{brown precipitate}} \right) \hfill \\ \end{gathered}$$

The working solution of the stain normally contains 60 mM sodium acetate, 5 mM sodium citrate, 4.7 mM copper (III) sulfate, 0.5 mM potassium ferricyanide, 1.7 mM acetylthiocholine iodide, and distilled water. However, since this composition failed to stain the embryos even after repeated trials and the use of freshly prepared solutions, we had to optimize the staining solution. Copper ions inhibit acetylcholinesterase and cause neurotoxicity in zebrafish [64]. Based on this, we hypothesized that decreasing the concentration of copper sulfate in the staining solution may remove or alleviate any inhibitory effect that it may have on the enzyme. We stained the embryos with staining solutions containing 4.7 mM (the original concentration in the Karnovsky method), 4.5 mM, and 4.3 mM of copper sulfate. No staining was observed in embryos treated with the solution containing 4.7 mM copper sulfate. Faint staining was observed with 4.5 mM copper sulfate. The expected intensity and pattern of staining were observed with 4.3 mM of copper sulfate. 6.8 g of sodium acetate, 12.905 g of sodium citrate, and 12.4845 g of cupric sulfate, each dissolved in 100 mL distilled water, 8.231 g of potassium ferricyanide in 50 ml distilled water, and 0.72295 g of acetylthiocholine iodide in 5 mL distilled water were used to prepare 0.5 M stock solutions for Karnovsky’s staining. 1 mL of paraformaldehyde (PFA) was used to fix 10 embryos in a vial. Before staining the embryos, PFA was removed from the vials. The embryos were washed with 1× PBS solution thrice for 5 min each. The embryos were then incubated in the stain. After 3 h, the stained embryos were washed thrice with 1× PBS-Tween (prepared by dissolving 140 µl of Tween 20 in 14 mL 1× PBS) and inspected under the microscope.

### Assessment of larval locomotor activity

The settings of wrMTrck, a freely available ImageJ plugin originally developed for examining multiple behavioral parameters in the nematode *Caenorhabditis elegans*, have been optimized in our lab for zebrafish larvae [43]. This software was used to study the locomotor patterns of treated larvae and calculate their average speed, distance, and body bend. Videos of 3 larvae swimming in a Petri dish were shot in a dark chamber by placing them on a light source. The videos were 3 min long; 2 min was allowed for acclimatization and 1 min for test response.

### Passive avoidance response test

Adult zebrafish treated with a test compound was transferred to an experimental chamber. This chamber consisted of a dark compartment and a lit compartment separated by a movable door. The fish was placed in the dark chamber and allowed to acclimatize for 3 min. After this, the door was opened. A stone was dropped in front of the fish 3 s after it crosses the door. The stone served as the shock stimulus in this scenario. Crossing time estimated as the time taken by the fish to cross the door from the moment the door was opened was recorded after every such ‘trial’. Three such trials made up ‘one training session’ for the acquisition of the avoidance response. Two hours after a training session, a ‘test session’ consisting of a single trial was conducted to assess the extent of learned avoidance response retention. The fish was exposed to the test compound in the experimental chamber during both sessions [35].

### Protein-small molecule rigid body docking

The crystallographic structure of human AChE was collected from the PDB database (PDB ID: 4PQE) [65]. 4PQE has been widely used as a model for human AChE in molecular docking studies [66–69]. Docking on human AChE (PDB ID: 4PQE) was performed using SwissDock [47]. Before docking, this structure was prepared—by repairing incomplete residues, deleting water molecules, adding hydrogen atoms, and assigning partial charges—using the ‘Dock Prep’ function in UCSF Chimera [70]. The 3D structures of the ligands were collected from the ZINC database [71], namely, acetylcholine (ZINC3079336), isoxazole (ZINC1420779), scopolamine (ZINC100196329), atropine (ZINC100009278), galantamine (ZINC491073), scopoletin/buxuletin (ZINC57733), and kaempferol (ZINC3869768). SwissDock generates binding poses of the ligands in the vicinity of target cavities and computes the summation of various types of energy. The docked poses of the ligands were visualized and curated using the ‘View Dock’ function in Chimera. Docked poses with minimal binding energy were chosen for further examination. Note that our AChE model (4PQE) neither had modified residues nor any ligands associated with it. We selected such a structure in order to perform an exploratory analysis with our ligands of interest, and detect different binding poses in all possible binding pockets within AChE. Despite employing the ‘blind docking’ approach, we were able to replicate key binding interactions, for example, (i) the interaction of ACh with Phe 295, which determines substrate specificity of the acyl pocket [72], and (ii) the binding of atropine and scopolamine—which are structurally identical—to the same residue (Glu 313). All the interactions reported in Figs. 6B–D, 7B–F and Table 1 fall within the range of hydrogen bonding limit (< 2.5 Å) [73].

## Supplementary Information

**Additional file 1: Figure S1.** Time spent by adult fishes in each of the three areas of the tank. Adult zebrafish treated with test compound(s) were transferred to an experimental chamber divided equally into three areas and the extent of lateralization in swimming was measured as time spent in each of the three areas (shown as histograms). Areas 1 and 3 are peripheral, whereas area 2 is central. The time spent in the three areas was determined by analyzing 3 min long videos. 2 min was allowed for acclimatization and 1 min for test response. The fish was either untreated or treated with scopolamine (SCOP), isoxazole (ISOX), and ISOX + SCOP. The fish was exposed to 200 µM of SCOP and/or 31.2 mM of ISOX.

## Data Availability

All data generated or analyzed during this study are included in this article.

## Abbreviations

ACh: Acetylcholine
AChE: Acetylcholinesterase
AD: Alzheimer’s disease
ATCh-I: Acetylthiocholine iodide
ChAT: Choline acetyltransferase
CP: *Convolvulus pluricaulis*
DTNB: 5,5ʹ-Dithiobis-(2-nitrobenzoic acid)
hpf: Hours post-fertilization
ISOX: Isoxazole
PBS: Phosphate buffer saline
PFA: Paraformaldehyde
SCOP: Scopolamine
ZFET: Zebrafish Embryo Toxicity Test
