# Supplementary figures and images for: Novel insights on acetylcholinesterase inhibition by Convolvuluspluricaulis, scopolamine and their combination in zebrafish
Source: Nat Prod Bioprospect. 2022 Feb 25;12(1):6. doi: 10.1007/s13659-022-00332-5 (PMC8881542; doi:10.1007/s13659-022-00332-5)

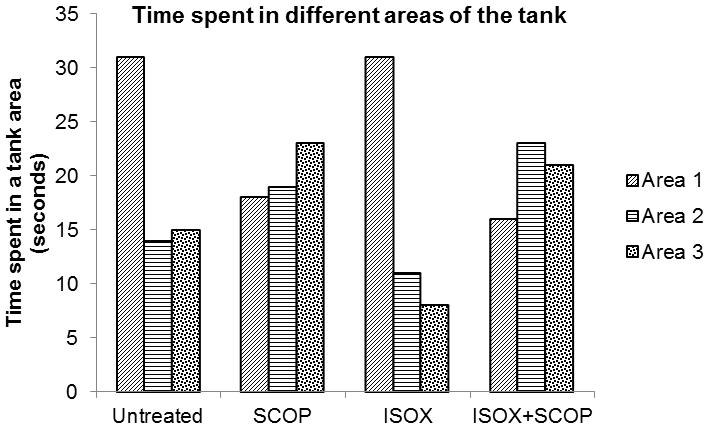

Supplement: Supplementary file 1 — Additional file 1: Figure S1. Time spent by adult fishes in each of the three areas of the tank. Adult zebrafish treated with test compound(s) were transferred to an experimental chamber divided equally into three areas and the extent of lateralization in swimming was measured as time spent in each of the three areas (shown as histograms). Areas 1 and 3 are peripheral, whereas area 2 is central. The time spent in the three areas was determined by analyzing 3 min long videos. 2 min was allowed for acclimatization and 1 min for test response. The fish was either untreated or treated with scopolamine (SCOP), isoxazole (ISOX), and ISOX + SCOP. The fish was exposed to 200 µM of SCOP and/or 31.2 mM of ISOX. [file 13659_2022_332_MOESM1_ESM.tif]
